# A Cross-Sectional Analysis of EMT Certification Rates among Navy Hospital Corpsmen in the INDO-PACIFIC

**DOI:** 10.12688/mep.20048.1

**Published:** 2024-05-31

**Authors:** Daniel Watson

**Affiliations:** 1 Operational Readiness Training, U.S. Navy Bureau of Medicine and Surgery, Falls Church, VA, 22042, USA

**Keywords:** EMT, Emergency Medical Technician, INDOPACIFIC, NREMT, National Registry, Hospital Corpsmen

## Abstract

**Introduction:**

Until 2017, the United States Navy (USN) incorporated Emergency Medical Technician (EMT) curriculum into Basic Medical Technician Corpsman (BMTC) training program. At overseas USN and Marine Corps installations, USN hospitals and clinics must train and certify EMTs to support the Emergency Medical System (EMS) mission. The primary aim of this study is to identify the impact of removing NREMT curriculum from BMTC on NREMT exam pass rates of students stationed in the Indo-Pacific (INDOPAC) region. This study examines and analyzes 1 ^st^ and 3 ^rd^ attempt NREMT exam pass rates of four OCONUS installations in the INDOPAC region over 10.5 years.

**Materials and methods:**

The researcher conducted a retrospective cross-sectional analysis of NREMT exam pass rates. Data were extracted from the NREMT database from four program sites in Japan. Five years of data before and after the curriculum change were included. Date ranges extracted were from January 1, 2012, to June 1, 2022.

**Results:**

During the observed 10.5 years, 1093 students attempted the NREMT cognitive examination within the INDOPAC region and were included. Cumulative pass rates for 1 ^st^ and 3 ^rd^ attempts for all four locations for all years were 64.2% and 71.1%, respectively. Comparison of the overall INDOPAC 1 ^st^ attempt pass rate before and after BMTC curriculum change shows a 62% pass rate for 2012–2017 (before EMT curriculum removal) and a 66% pass rate for 2017-2022 (after removal). The two means do not show statistical significance as the p-value is determined to be 0.172 (P>0.05).

**Conclusions:**

No statistical correlation between students before and after the curriculum change was found. A correlation was identified between pass rates and delayed examination, indicating if the national exam is delayed, the rate of certification decreases.

## Introduction

Emergency Medical Technicians (EMTs) are nationally certified healthcare professionals who provide emergency pre-hospital medical care in various pre-hospital environments. EMTs are first responders trained to perform patient assessments and apply life-saving care at the point of injury and in an ambulance during patient transport to a higher echelon of care. The medical care provided in this setting can vary widely and include life-saving interventions for emergencies such as strokes, heart attacks, respiratory distress, and physical injuries resulting from trauma ^1^. To ensure first responders are competent to assess and perform medical interventions in the pre-hospital setting, The National Registry established National standards for four levels of pre-hospital responder certification: Emergency Medical Responder, EMT, Advanced Emergency Medical Technician, and Paramedic ^2^. EMT is the most common pre-hospital certification held by first responders within the United States. Licensure is granted at the State level after obtaining certification by passing the National Registry Exam. Like the civilian sector, historically, the pre-hospital portion of an overarching Emergency Medical System (EMS) on military bases is performed by service members who are EMT certified. To obtain the national certification, students must attend and pass a National Registry of Emergency Medical Technicians (NREMT) approved training course and pass the NREMT exam. US military bases are considered federal land or US-occupied regardless of location. For both continental United States (CONUS) and outside the continental United States (OCONUS) US Navy (USN) bases, the National Registry has granted the USN “Navy State” status, which allows the USN to teach NREMT programs without boundaries.

From 2008 to the present, policy and educational system changes have led to an absence of an EMT-producing pipeline, diminishing the inventory of EMTs, and left OCONUS USN and Marine Corps (MC) installations to train and certify their own EMTs to perform pre-hospital response. Serving as the initial policy change in 2008 a USN instruction directed the Bureau of Medicine and Surgery (BUMED) to transfer EMS responsibilities including pre-hospital care to Commander, Naval Installations Command (CNIC) for stateside USN and MC installations but excluded OCONUS installations ^3^.

There are two EMT training systems of interest within the USN to deliver EMT training to USN students leading to NREMT certification. The first system teaches and certifies Hospital Corpsmen (HM) at the initial entry point after USN basic training–at HM School–serving as a training and certification pipeline to OCONUS installations. The second relies upon OCONUS commands to train and certify EMTs upon arrival. These two systems respond and rely heavily on the EMS mission and policy CONUS and OCONUS.

The first training system is located at San Antonio, Texas, at the Medical Education Training Campus (METC). Historically, USN, US Air Force (USAF), and US Army’s (USA) basic medical training has been independent and separate, and training locations have independently changed; however, in 2011, all three services relocated to METC at Joint Base San Antonio, Texas. The Services aligned USN and USAF curriculums to establish a standard knowledge base and was referred to as Basic Medical Technician Corpsman (BMTC) Program. For most of the program, USN and USAF were taught together at METC, including the NREMT curriculum and certification ^4, 5^. Even though the USA was on the same campus, they maintained their enlisted medical program and curriculum separate from USN and USAF’s BMTC. Both BMTC and the USA's training courses contained NREMT curriculum, and their programs allowed students from all three Services to attempt NREMT certification after completing their program ^4, 5^. USA and USAF medics were required to obtain certification and currently remain a condition of employment ^5^. This requirement mandates students of the two services attempt and pass the NREMT exam to achieve NREMT certification to graduate and hold the medic job rating. In juxtaposition, the USN did not and currently does not require HMs to attempt the NREMT exam nor obtain certification; however, after BMTC graduation and before departing METC, HMs were provided the opportunity, without penalty for failure, to attempt the NREMT exam.

After six years of consolidation and establishment of BMTC, Naval Medical Forces Support Command (formerly Navy Medicine Education and Training Command) intended to de-consolidate from the USAF and the BMTC curriculum and establish a separate curriculum for USN students as stated in an internal memorandum to BUMED dated October 13, 2016. In the same year, BUMED issued an Instruction indicating that ranks E5 and below HMs who transfer to OCONUS locations without EMT training programs will receive training CONUS before being transferred, however, the execution of this policy did not occur ^6^. The de-consolidation removed five weeks of dedicated NREMT curriculum and added curriculum elements found to be more aligned with HM expeditionary medical job tasks due to the findings of an HM job duty task analysis. The resulting curriculum change occurred in 2017. This curriculum change eliminated the opportunity for HMs to attempt the NREMT exam at METC while dissolving the only EMT-producing pipeline that feeds trained and certified EMTs to all USN installations, including OCONUS installations. Since 2017, USN OCONUS Military Treatment Facilities (MTFs) remain required to organically produce enough certified EMTs to sustain their critical pre-hospital ambulance services throughout the Indo-Pacific (INDOPAC) region. As a mitigation strategy for the most remote Navy Installations with pre-hospital EMS, Chief, BUMED issued a memorandum on April 8, 2019, requesting Commander Navy Personnel Command to add an Intermediate Stop (I-STOP) to permanent change of station orders for HMs to attend an EMT course and certification in Yokosuka, Japan. In 2020, the USN directed BUMED to coordinate the transfer of EMS to CNIC ^7^.

**Figure 1.**
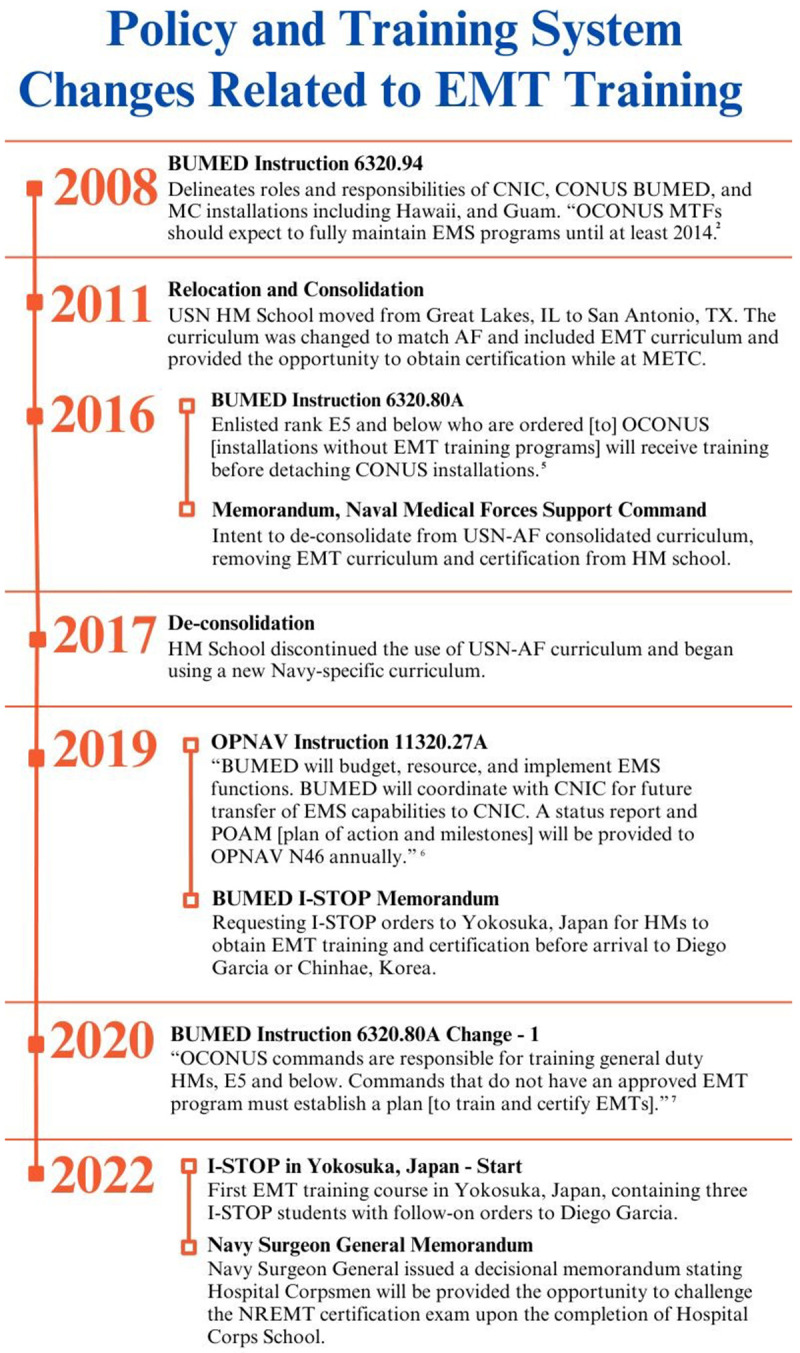
Policy and training system changes related to EMT training. CNIC – Commander Navy Installation Command, CONUS – Continental United States, BUMED – Bureau of Medicine and Surgery, MTF – Military Treatment Facility, USN – United States Navy, AF – Air Force, OPNAV EMT – Emergency Medical Technician, STOP – Intermediate Stop, NREMT – National Registry of Emergency Medical Technicians.

In the years 2020 and 2021, Chief BUMED updated existing guidance for OCONUS commands, placing the responsibility to train HMs identified to work in the emergency department on the receiving OCONUS command and subsequently issued a decisional memorandum to Navy Medicine dated August 4, 2022, directing Navy Medicine to provide HMs the opportunity to challenge the NREMT exam upon completion of HM School ^8^. The 2020 BUMED guidance implied OCONUS commands would train and certify their own HMs as EMTs, while the 2021 decisional memorandum assured OCONUS commands that a portion of HMs would arrive EMT-certified or, at minimum if the HM’s certification attempt were unsuccessful at METC, would qualify them for an EMT refresher course upon arrival OCONUS. If HMs were indeed given the chance to take the national exam before departing METC the resulting benefit to OCONUS commands would likely be an increased quantity of HMs who arrive trained and certified to support the EMS mission. The current HM school curriculum was not evaluated as part of this study. However, the inclusion of an NREMT exam at course completion would likely necessitate a curriculum revision or, at minimum, direct an emphasis on specific pre-hospital skills already within the current curriculum to meet the NREMT program standard. Figure 1 illustrates and summarizes the timeline of events from 2008 until the present as described herein.

At the time of this study, CNIC has yet to assume responsibility for pre-hospital EMS at OCONUS USN and MC installations, nor has an EMT training pipeline for HMs who are ordered to OCONUS USN and MC installations been formally established – obligating OCONUS USN Commands to train, certify, and maintain pre-hospital EMS. Policy and directives, directly or indirectly, addressing a CONUS EMT pipeline to support OCONUS USN and MC installations have been published, however, the failure to execute policy has prevented mild to complete resolution, leaving OCONUS Commands to problem-solve at the local level. As OCONUS installations teach EMT training courses they are the sole beneficiaries of their certification pass rate, so maintaining high enrollment and pass rate is imperative to maintain an adequate number of EMTs. The transient nature of military personnel on OCONUS orders inflicts a high staff turnover rate for EMT program directors, instructors, subject matter experts, and certified EMTs who all contribute to the continuity or discontinuity of the training course. Due to the exact transient nature, trained and certified EMTs rotate to other installations quickly and must be timely replaced by training and certifying another EMT.

This study assesses the impact of the 2017 BMTC de-consolidation that removed EMT training and certification on certification pass rates at OCONUS installations. Considering the unexecuted string of policies that could have removed or lessened the OCONUS EMS responsibility and added a CONUS EMT pipeline, this study looks at past and present EMT certification pass rates to explore a possible OCONUS EMS benefit if EMT training and certification was incorporated back into HM School. As an emphasis on pre-hospital patient care was replaced by operational-centric modules at HM School, the researcher asks if the challenge to train and certify EMTs once they arrive OCONUS is lessened, more challenging, or if it remains the same. As OCONUS commands struggle to train and certify enough EMTs to meet their pre-hospital EMS mission in an already resource-constrained environment how can policy and resource allocation shift or find a balance between the two training systems the USN uses to meet the OCONUS pre-hospital EMS mission? It is also unknown if the USN’s reluctance to mandate EMT certification before graduation from HM School plays a role in the USN’s pass rate. Since the impact of the 2017 curriculum change on the quantity of EMTs either CONUS or OCONUS has not been evaluated, the results of this study can, perhaps, affect positive change in the effort to train and certify more EMTs. The vacillation shown in the policy and training systems in Figure 1 likely reflects the transient nature of senior leadership and decisions made in the absence of data and research on this subject. Adding to its complexity, OCONUS hospitals have transitioned patient care delivery within the MTF from BUMED to Defense Health Agency (DHA), however, DHA does not manage or own the pre-hospital portion of the EMS mission. This leaves the critical pre-hospital EMS responsibility with BUMED as CNIC has not assumed EMS at OCONUS locations. A significant concern among OCONUS hospitals is the ability to certify enough EMTs to the national standard to safely sustain the enduring EMS mission, regardless of which Department of Defense entity maintains responsibility. These questions were not answered by previous, similar studies. Several studies examined NREMT pass rates for all three services in 2017 ^4, 5^. No studies have been published examining USN certification pass rates after the 2017 BMET deconsolidation at METC, generally or in one geographic region. Previous studies compared USA and USAF medics and USN HMs certification pass rates ^4, 5^. They reported that the pass rate for USN HMs was approximately 30% lower and theorized the USA and USAF’s mandate to attempt the exam and achieve certification was a primary influence ^4, 5^. Studies have also looked at exam pass rates and their relationship to size and geographic location but specifically eliminated military students from their population ^9^.

This study was conducted in 2022 during the last four months of the researcher's tour at Naval Medical Readiness and Training Command (NMRTC) Yokosuka, Japan. After overseeing an NREMT program that produced unpredictable and varying pass rates, the researcher collected historical to present-day national exam data from local and neighboring EMT training sites within the region to assess for trends and correlations that would be helpful to OCONUS USN commands that run EMT training sites and influence policy and resource allocation.

The primary aim of this study is to identify, if any, the impact of removing NREMT curriculum from HM School on NREMT exam pass rates of students stationed in the INDOPAC region. The secondary aim is to identify national exam pass and fail trends between course completion and exam date. The secondary aim was derived from the researcher’s observation of course and exam dates that were supplemental and organic to the data query output. The researcher found the supplemental data relevant to share with EMT training site managers. This study will examine and analyze 1 ^st^ and 3 ^rd^ attempt NREMT exam pass rates of four OCONUS EMT training sites in the INDOPAC region. Supplemental data and analysis were used to compare program site pass dates to national exam pass rates, total certified EMTs in each INDOPAC location, and assess for correlation between pass rates and time elapsed between the end of the course and certification exam attempt. However warranted, due to the variability in programs, including various factors that affect the continuity of EMT training programs at OCONUS sites, this study doesn't lead itself as an evaluation of an EMT program as a whole or of one location.

## Methods

This retrospective review of certification pass rates was determined not to be human-subject research by Navy Medicine and Readiness Command Yokosuka leadership and was cleared for release by USN Bureau of Medicine and Surgery.

**Figure 2.**
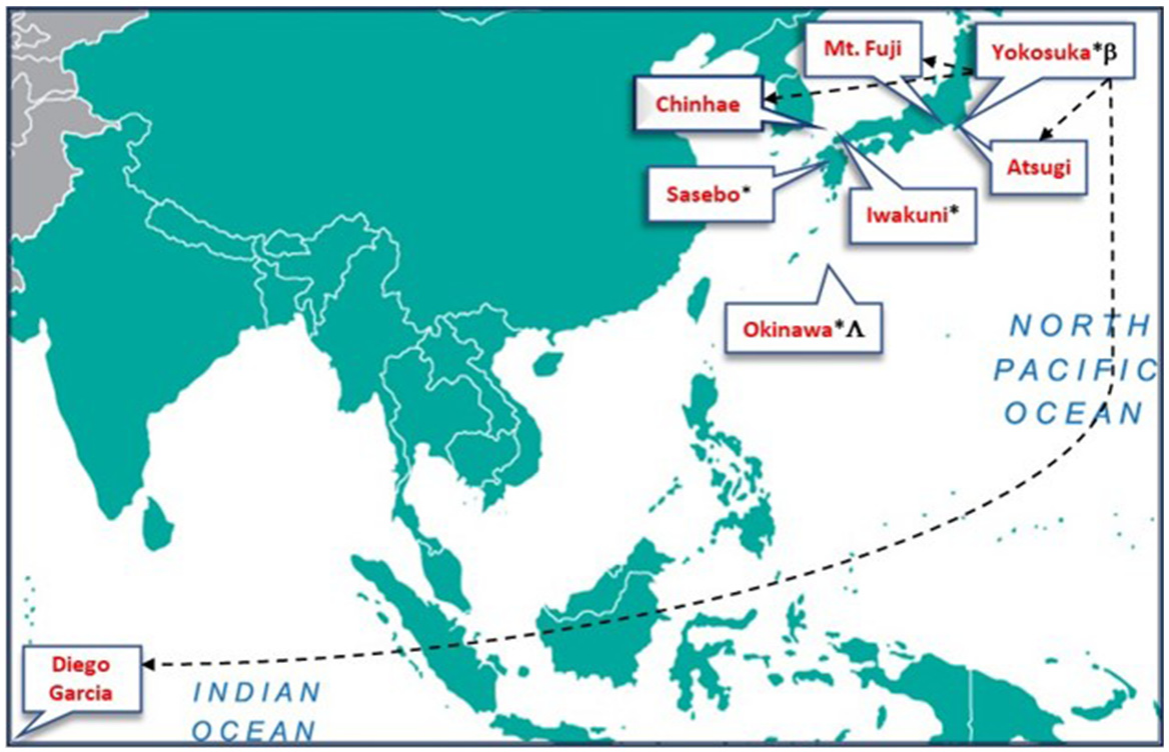
U.S. Navy medical treatment facilities within the Indo-Pacific where USN Hospital Corpsmen perform as Emergency Medical Technicians. * EMT training and certification site location. Ʌ Provides EMT course instruction and certification to 5 additional sites in Okinawa, Japan. ß Provides EMT course instruction and certification to students from sites without EMT training programs. Atsugi, and Camp Fuji in Japan, Diego Garcia, and Chinhae, South Korea. 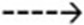 Shows flow of EMTs after training and certification in Yokosuka, Japan.

The researcher conducted a retrospective cross-sectional analysis of NREMT exam pass rates. The study design was chosen to make a direct comparison of data influenced by an unmanipulated variable and collated at a fixed point in time. Data were extracted from the NREMT database ^10, 11^ from four program sites in Japan. A convenience sample of two sites on MC installations in Iwakuni and Okinawa, Japan, and two on USN installations in Yokosuka and Sasebo, Japan ( Figure 2) were included. These sites were selected for inclusion as they contain similar populations and resources in addition to being a convenience sample based on the researcher’s location and access to data. Approximately five years of data were available after the curriculum change in 2017, so five years before the event was selected for inclusion to draw contrast and act as a control. EMT program directors at each site were provided a standardized set of instructions detailing a standard query process within the NREMT database to ensure the reliability of the raw output of each site. Date ranges extracted were from January 1, 2012, to June 1, 2022. They included raw NREMT exam data for all students who attempted the NREMT exam under their program's account, course completion date, attempt number, and exam date. Raw site data were consolidated in Microsoft Excel and imported into Statistical Package for the Social Sciences (SPSS) 27. Before descriptive statistics were applied, 4 ^th^, 5 ^th^, and 6 ^th^ attempts were excluded from the raw data, and student names were replaced by a randomized number. National pass rates per year were either extracted from annual reports ^10, 12^ or by following the exact standardized instructions used to pull student exam data, except the "program" option was replaced by selecting "national" as the report type. 4 ^th^ and subsequent exam attempts were excluded due to the number of students who attempted the exam greater than three times was so minimal that conclusions could not be drawn from the data.

### Measures

Measures of data for exam pass rates are expressed as 1 ^st^ and cumulative 3 ^rd^ attempts. 1 ^st^ attempt pass rate represents students who pass the NREMT exam on their first attempt. The third exam pass rate represents students who passed on 1 ^st^ 2 ^nd^ or 3 ^rd^ attempt and is defined as the final pass rate by the researcher. Not all students who failed their first exam attempted the exam a 2 ^nd^ time or 3 ^rd^ time. National pass rates ^10^ represent data from all EMT programs under the National Registry, including the four sites studied. Any time elapsed greater than 30 days was considered delayed. The National Registry does not define or recommend an interval between the end of the course and attempting the certification exam, however, the researcher considered a reasonable amount of time for students to complete the administrative intensive process for a student to apply for exam funding and apply for the exam on NREMT’s website was less than 30 days. Delays in taking the exam were measured by calendar days elapsed between the end of the course and the exam date, as shown on NREMT program data extraction reports. Quantitative data showing total certifications were reported as pulled from the NREMT database.

### Analysis

Comparison of pass rates between the two timeframes of interest was assessed for significance using Levene's Test for Equality of Variances and t-test for Equality of Means.

Comparisons of pass rates between the number of days between the course completion and exam date were made using Pearson Correlation. Statistical significance was determined using a p-value of 0.05 and an alpha of 0.05 to determine a 95% confidence level. All analyses were carried out using SPSS.

## Results

During the observed 10.5 years of exam data, 1093 USN students completed an EMT-level program and attempted the NREMT cognitive examination at one of the four included sites within the INDOPAC region. A total of 702 students passed during their first attempt. Of the 391 who did not pass their first attempt, only 45% (n=176) attempted a 2 ^nd^ exam, and out of 177 who did not pass their 2 ^nd^ attempt, only 27.6% (n=49) attempted a 3 ^rd^ exam. Only 17 students passed their 3 ^rd^ attempt. Cumulative pass rates for 1 ^st^ and 3 ^rd^ attempts for all four INDOPAC locations for all years were 64.2% and 71.1%, respectively ( Table 1). Total EMTs certified (by 3 ^rd^ attempt) by site were shown to be relative to Installation size: Yokosuka 280, Okinawa 272, Iwakuni 127, Sasebo 99. Overall, 778 EMTs were ultimately certified by their 3 ^rd^ attempt.

**Table 1.** National Registry of Emergency Medical Technicians (NREMT) Cognitive Exam 1 ^st^ and cumulative 3 ^rd^ attempts pass rates. Indo - Pacific *versus* national average.

***1 ^st^ Attempt***	*2012*	*2013*	*2014*	*2015*	*2016*	*2017*	*2018*	*2019*	*2020*	*2021*	*2022* *
*Indo-Pacific*	62%	74%	61%	72%	53%	56%	65%	65%	71%	67%	75%
*national average*	70%	68%	67%	66%	68%	68%	68%	68%	66%	67%	66%
***3 ^rd^ Attempt***											
*Indo-Pacific*	65%	77%	70%	76%	65%	63%	70%	76%	76%	79%	79%
*national average*	81%	80%	79%	79%	80%	81%	74%	73%	79%	79%	78%

*Data query for 2022 excludes June 2, 2022 – December 31, 2022.

**Figure 3.**
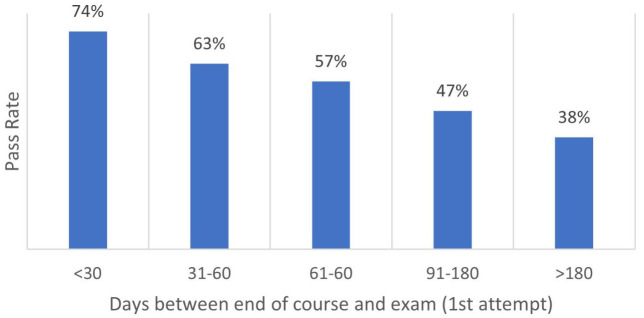
Correlation between EMT course completion and examination date.

Comparison of the overall INDOPAC 1 ^st^ attempt pass rate before and after HM School curriculum change shows a 62% pass rate for 2012-2017 (before EMT curriculum removal) and a 66% pass rate for 2017–2022 (after removal). The two means do not show statistical significance as the p-value is determined to be 0.172 (P>0.05). The correlation between course completion and exam date is significant at the 0.01 level (2-tailed), suggesting the longer the exam is delayed after course completion, the lower the 1 ^st^ exam pass rate will be ( Figure 3).

## Discussion

This study examined a specific population’s performance on the NREMT certification exam and attempted to find a correlation between students who received initial exposure and training on pre-hospital care during HM School and exam performance at follow-on OCONUS installations. The results of the measured and analyzed data could not show a significant statistical difference in pass rates. Since there was no statistical difference found between pass rates before and after the curriculum change, it suggests a re-introduction of full EMT curriculum at HM School would not produce better pass rates for HMs at follow-on OCONUS commands alone as the only intervention. What remains unknown is how the opportunity to certify at METC increases the overall quantity of EMTs Navy-wide and if the increased quantity reduces the OCONUS burden to train and certify HMs to support their EMS mission. Examining pass rates of OCONUS program sites becomes significantly less important to the success of the OCONUS EMS mission only if HM School teaches the EMT curriculum and administers the certification exam. Both teaching EMT curriculum and administering the certification exam at the same location prevents a delay in examination and may increase the pass rate. One such course of action to facilitate both teaching and examination proposed at the regional level is adopting a CONUS I-STOP that provides training and certification after METC and *en route* to OCONUS locations and would quality as the 3rd training system option not yet explored.

There is no existing literature that has measured and analyzed Navy State data at OCONUS installations neither before nor after HM School curriculum changed and its effect on future certifications. Baseline pass rates for Hospital Corpsmen at HM School for three years were examined by prior researchers. They measured pass rates of USN students who attempted certification while at HM School in the years 2015, 2016, and 2017 and found the USN pass rate for first attempt was 41%, 56%, and 62%, respectively ^4^.

As a general self-assessment performance marker, programs often compare their pass rate against the national average. Table 1 compared INDOPAC pass rate to the national average over 10 years. By the 3 ^rd^ attempt, INDOPAC’s pass rate only exceeded the national average twice (2019 and 2022) and matched the national average once (2021). The researcher identified several factors that may influence pass rates in INDOPAC that are unique to the student population. While the researcher was discussing the EMT program with Navy EMT leadership, one leader reported that since passing the NREMT exam resulted in the EMT working a 24-hour EMT duty, it was suspected that some students may purposely fail the exam to avoid certification (Personal communication). This remains unverified and would be difficult to prove retrospectively or presently. However, it remains one reason site leaders keep enrollment in an EMT course voluntary at all locations albeit this method admittedly reduces student enrollment and ability to produce high numbers of EMTs. The researcher found this reported hearsay necessary for inclusion as it is unique to the USN so long as certification remains unbound to employment and related to extra duty. 55% (n=215) of students who failed their first exam did not attempt re-examination for unknown reasons. The civilian sector re-examination attempt is significantly higher as found in a study of non-military EMT students where only 33% of students who were eligible for re-examination did not attempt the NREMT a second time ^13^. Research into barriers to re-examination for the civilian sector has been unexplored ^14^. Future qualitative or mixed studies on the low re-examination rate of HMs would benefit EMT program administrators, pass rate, and EMT inventory in both sectors.

Even though this study was not conducted to provide program management strategies, the researcher found responsibility in statistically analyzing and reporting a correlation between course completion and the exam date. When observing the program report data, it was evident that one factor might influence the pass rate: delayed examination. The researcher, a prior EMT and EMT Program Director, has both experienced and had discussions with current EMT program site directors about the administrative and technical hurdles students and directors must navigate to obtain exam funding and scheduling. The leadership and oversight change compounds these hurdles as the students are moved back to their assigned department within the MTF and are no longer under the control of the EMT Program Director. This data supports the preliminary conclusion that if HMs are taught the EMT curriculum at HM School but do not test before they leave METC, the delay in examination significantly reduces their chance of obtaining certification. The reported administrative hurdles for a student to take the exam can prevent students from pursuing the exam independently at METC or after departure. The “opportunity” alone will not certify HMs as EMTs, alternatively, the administrative navigation leading a student to certification should be programmed into the course schedule.

This research was conducted from resulting downstream effects of a training curriculum change and various EMS and EMT policies and directives that have remained unexecuted. Admittedly, the results of this study alone likely do not provide Navy Medicine leadership with enough information to forge a clear path to address the foundational problem of a low EMT inventory. However, along with the results, this study provides an account of the events that could have provided and remain viable COAS using the two EMT training systems or a third system; a CONUS EMT training I-STOP outside of HM School, or a mixture of the three.

## Limitations

This study's findings are limited to its retrospective design and the absence of detailed student demographics. Even though this study found a correlation in one of its data analyses, retrospective studies cannot determine causation. Its retrospective data capture method contains several unknowns. Even though it was reported and observed that recent students were made up of USN HMs, it is possible in past courses that some of the students within the data were not USN HMs. Since these four Navy State EMT programs were the only programs in the INDOPAC region, it is probable some students represented in the data were United States Marines, non-HM rates, or U.S. government civilians. It is also unknown if some exam scores were recertifications. Student demographic data could not be extracted from the NREMT website with current permissions that would have provided helpful insight. The enlisted rank of each student, career experience that helped or hindered success, or age of students remain unknown. Program staffing, resources, and instructor quality were not part of this study and should not be assumed to be either poor or robust, nor is there evidence that suggests their impact on student pass rate.

## Conclusions

This study's aim and primary objective was to analyze NREMT exam pass rates in the INDOPAC region before and after HM curriculum was changed at METC in 2017. No statistical correlation between students before and after the change was found. A correlation was identified between pass rates and delayed examination, indicating when the national exam is delayed the rate of certification decreases, which should be shared with program administrators and students to adapt local process to facilitate students to the exam within 30 days. Deeper mining of quantitative data from program sites and students who were successful and unsuccessful would highlight program site strengths and weaknesses and produce valuable lessons learned to apply to future courses and such data would lend itself to a program evaluation study. Further studies on the feasibility of OCONUS sites to self-sustain pre-hospital EMS, comparison of the three training systems, and implementation of an EMT pipeline that trains and certifies HMs before OCONUS arrival, and identifying barriers to retesting would be impactful to the OCONUS EMS mission.

## Ethics and consent

Ethical approval and consent were not required.

## Data Availability

Figshare: RAW NREMT Exam Data Japan Training Sites ^11^. https://doi.org/10.6084/m9.figshare.24598539. The project contains the following underlying data: Raw_data_SPSS (1).xlsx Data are available under the terms of the Creative Commons Attribution 4.0 International license (CC-BY 4.0).
