# Supplementary material for: A Cross-Sectional Analysis of EMT Certification Rates among Navy Hospital Corpsmen in the INDO-PACIFIC
Source: MedEdPublish (2016). 2024 May 31;14:41. [Version 1] doi: 10.12688/mep.20048.1 (PMC11322699; doi:10.12688/mep.20048.1)
Supplement: Peer review history

# Reviewer report: Reviewer response for version 1

**DOI:** 10.21956/mep.21466.r37878

**Published:** 2024-08-13

**Authors:** Gerard Bury

**Affiliations:** 1 University College Dublin, Dublin, Leinster, Ireland

An interesting and potentially useful contribution to the literature on the impact of organisational systems on individual student performance in end-point exams.  A number of issues arise:

 1. The text concludes by saying that 'ethics permission is not required'.  It's not at all clear that this is the case and I would ask that the journal editor make a decision on the issue.  Permission to access performance data at four testing centres was given by the (presumably) owner military body but this is not the same as research ethics approval.  It would appear from the text that data gatherers working on behalf of the author were asked to collate student data 'and student names were replaced by a randomized number'.  This strongly suggests uses of identifiable student exam data (and apparently without student permission) which certainly is a standard for research ethics approval.

 2. The introduction is very long and much of the chronology is summarised in the associated table.  The text of the introduction could be considerably shortened and re-focused to the author's critique of the military systems' changes in policy on EMT training.  Although comprehensively outlined, the reasons for the many changes described are not addressed, which would be interesting as context.  

 3. For a civilian and European audience, the many military acronyms might be reduced?  What is COAS?

 4. Data from four training / testing sites in the Far East are used and described as a convenience sample; however, later in the text, they are described as the only sites - please clarify.  If a convenience sample - of how many others and on what basis were they chosen / others excluded?

 5. The study itself is a before / after comparison of pass rates over two five year periods, showing little difference between periods.  The limitations should perhaps explore other factors such as the ongoing conflicts in the earlier period, the impact of COVID in the later period - and also acknowledge that many other issues may also be relevant.

 6. The discussion includes a number of hearsay / personal experience comments on the findings with limited reference to the literature on the effect of institutional policy / change on student performance.  Both aspects should be re-considered - particularly if some of the commentary may be perceived as criticism of students, without evidence  contained in the study.

 7. The study has the potential to highlight important systems issues - consideration of the above issues may be of value.

Have any limitations of the research been acknowledged?

Yes

Is the work clearly and accurately presented and does it cite the current literature?

No

If applicable, is the statistical analysis and its interpretation appropriate?

Yes

Are all the source data underlying the results available to ensure full reproducibility?

No

Is the study design appropriate and is the work technically sound?

Yes

Are the conclusions drawn adequately supported by the results?

Partly

Are sufficient details of methods and analysis provided to allow replication by others?

Yes

Reviewer Expertise:

Pre-hospital emergency care

I confirm that I have read this submission and believe that I have an appropriate level of expertise to confirm that it is of an acceptable scientific standard.

# Reviewer report: Reviewer response for version 1

**DOI:** 10.21956/mep.21466.r38039

**Published:** 2024-08-08

**Authors:** Jeffrey Lubin

**Affiliations:** 1 Penn State College of Medicine, Hershey, USA

The researcher conducted a retrospective analysis of NREMT exam pass rates at U.S. Navy medical treatment facilities in the Indo-Pacific region. To draw contrasts, approximately five years of data after the 2017 curriculum change were compared to five years before the event. The study suggested that the change in curriculum did change pass rates on the NREMT exam and highlighted a correlation demonstrating that delayed examination after course completion significantly reduces the chance of obtaining certification. 

**Overall, the article is well written. A few suggestions:**

- The introduction is extremely long and could benefit from editing.
- The data point of 1st attempt pass rate makes sense, but the term 3rd attempt pass rate is confusing. For half of the article, I was wondering why the 2nd attempt wasn't included.  Perhaps it would be better to re-phrase as "passing by the 3rd attempt" and then using an abbreviation (P3A or something like that). 
- The author mentions the delayed examination finding in the abstract, but there is nothing in the results section of the abstract to support that (even if there is in the full article). 
- In the measures section, the author mentions that he felt a "reasonable amount of time" to complete the exam was 30 days. What is this based on?

Have any limitations of the research been acknowledged?

Yes

Is the work clearly and accurately presented and does it cite the current literature?

Yes

If applicable, is the statistical analysis and its interpretation appropriate?

Yes

Are all the source data underlying the results available to ensure full reproducibility?

Yes

Is the study design appropriate and is the work technically sound?

Yes

Are the conclusions drawn adequately supported by the results?

Yes

Are sufficient details of methods and analysis provided to allow replication by others?

Yes

Reviewer Expertise:

Emergency Medicine, Emergency Medical Services

I confirm that I have read this submission and believe that I have an appropriate level of expertise to confirm that it is of an acceptable scientific standard.
